# Predicting 6-Month Unfavorable Outcome of Acute Ischemic Stroke Using Machine Learning

**DOI:** 10.3389/fneur.2020.539509

**Published:** 2020-11-19

**Authors:** Xiang Li, XiDing Pan, ChunLian Jiang, MingRu Wu, YuKai Liu, FuSang Wang, XiaoHan Zheng, Jie Yang, Chao Sun, YuBing Zhu, JunShan Zhou, ShiHao Wang, Zheng Zhao, JianJun Zou

**Affiliations:** ^1^School of Basic Medicine and Clinical Pharmacy, China Pharmaceutical University, Nanjing, China; ^2^Department of Clinical Pharmacology, Nanjing First Hospital, Nanjing Medical University, Nanjing, China; ^3^Department of Pharmacy, Nanjing First Hospital, Nanjing Medical University, Nanjing, China; ^4^Department of Pathology, Nanjing First Hospital, Nanjing Medical University, Nanjing, China; ^5^Department of Neurology, Nanjing First Hospital, Nanjing Medical University, Nanjing, China; ^6^Department of Neurology, the First Affiliated Hospital of Chengdu Medical College, Chengdu, China; ^7^School of Public Health, Bengbu Medical College, Bengbu, China

**Keywords:** cerebral ischemia, machine learning, prediction, unfavorable outcome, stroke

## Abstract

**Background and Purpose:** Accurate prediction of functional outcome after stroke would provide evidence for reasonable post-stroke management. This study aimed to develop a machine learning-based prediction model for 6-month unfavorable functional outcome in Chinese acute ischemic stroke (AIS) patient.

**Methods:** We collected AIS patients at National Advanced Stroke Center of Nanjing First Hospital (China) between September 2016 and March 2019. The unfavorable outcome was defined as modified Rankin Scale score (mRS) 3–6 at 6-month. We developed five machine-learning models (logistic regression, support vector machine, random forest classifier, extreme gradient boosting, and fully-connected deep neural network) and assessed the discriminative performance by the area under the receiver-operating characteristic curve. We also compared them to the Houston Intra-arterial Recanalization Therapy (HIAT) score, the Totaled Health Risks in Vascular Events (THRIVE) score, and the NADE nomogram.

**Results:** A total of 1,735 patients were included into this study, and 541 (31.2%) of them had unfavorable outcomes. Incorporating age, National Institutes of Health Stroke Scale score at admission, premorbid mRS, fasting blood glucose, and creatinine, there were similar predictive performance between our machine-learning models, while they are significantly better than HIAT score, THRIVE score, and NADE nomogram.

**Conclusions:** Compared with the HIAT score, the THRIVE score, and the NADE nomogram, the RFC model can improve the prediction of 6-month outcome in Chinese AIS patients.

## Introduction

Globally, stroke is a leading cause of mortality and disability (1). In developing countries, the prevalence of stroke is increasing as the population ages. Patients who survive stroke have an increased economic burden due to post-stroke care (2). Therefore, accurate prediction of functional outcome after stroke would provide evidence for reasonable post-stroke management and thus improve the allocation of health care resources.

The prognostic prediction requires the processing of patients' clinical data, such as demographic information, clinical features, and laboratory tests results. Then, the model is developed to predict prognosis base on existing data. Several prognostic models have been developed to predict the clinical outcome after stroke, such as Houston Intra-arterial Recanalization Therapy (HIAT) score, Totaled Health Risks in Vascular Events (THRIVE) score and NADE nomogram (3–5). They are generally based on regression model with the assumption of a linear relationship between variables and the outcomes. The THRIVE score and HIAT score were developed based on Whites or Blacks, not Asians. Compared with White patients, the average age of Asian patients was younger (6, 7). In addition, several studies have observed worse survival in Whites with stroke compare to other race (8, 9). Importantly, the long-term outcomes of stroke were significantly different by race (7). Thus, it is difficult for these models to achieve accurate predictive performances on the Chinese population.

Machine-learning (ML) approaches have been widely used in medical fields (10). Recently, it has shown effective capability in disease prediction, especially in the analysis of large datasets with a multitude of variables (11–13). ML uses computer algorithms to build a model from labeled data and to make data-driven predictions. It enables the computer to process complex non-linear relationships between variables and outcomes, which may be hard to be detected by conventional regression models (14). Such advantages increase the accuracy of prediction model. ML includes multiple algorithms, such as logistic regression (LR), random forest classifier (RFC), support vector machine (SVM), fully-connected deep neural network (DNN), and extreme gradient boosting (XGBoost). The optimal selection of algorithm should be in accordance with the characteristics of the dataset. Meanwhile, the popularity of electronic patient record (EPR) systems and wide availability of structured patient data make sophisticated computer algorithms implemented at the bedside a reality.

In this study, we aim to develop the models using ML method to predict 6-month unfavorable outcomes in Chinese stroke patients, and then compare the performance of ML-based methods with existing clinical prediction scores.

## Methods

### Study Population and Clinical Baseline Characteristic

We retrospectively conducted an analysis using a cohort of acute ischemic stroke (AIS) patients who were admitted within 7 days of the onset of symptoms. The cohort included 3,231 consecutive AIS patients admitted at National Advanced Stroke Center of Nanjing First Hospital (China) between September 2016 and March 2019. The exclusion criteria were patients with missing data on pretreatment variables or long-term clinical outcome, signs of intracranial hemorrhage on baseline brain computed tomography scan, age < 18 years. We discarded all variables with 25% missing values or more for further analysis.

All clinical, anamnestic, and demographic characteristics were recorded at the time of admission, including the following data: age, sex, body mass index, National Institute of Health stroke scale (NIHSS) at admission, premorbid modified Rankin Scale (mRS), interval from onset to hospital within 4.5 h, systolic blood pressure, diastolic blood pressure, platelet count, urea nitrogen, creatinine, fasting blood glucose (FBG), and medical history such as hypertension, previous cerebral infarction, and so on. NIHSS at admission and premorbid mRS were presented as continuous variables in all models to increase model efficiency, and the ordinal scores were assumed to be linear. Unfavorable outcome was defined as mRS 3–6, 6 months after stroke. During face-to-face or *via* telephone follow-up with the patients using structured interview, their relatives or their general practitioners, certified neurologists, evaluated the baseline NIHSS and mRS scores.

### Statistical Analysis

The AIS patients were randomly stratified (8:2) into the training set for developing models, and the testing was set for evaluating the models' performance, which meant that the sampling was in proportion to the original dataset. We initially compared the clinical characteristics of patients with 6-month favorable and unfavorable outcomes in the training set. Continuous variables were reported as median value and interquartile range, and the various groups were explored for differences using the Mann–Whitney *U-*test. Categorical variables were instead expressed as number of events and percentage, dividing the number of events by the total number excluding missing and unknown cases. To compare categorical variables, Fisher's exact test or the χ^2^ test were used. To identify which variables were independently associated with poor outcome, all potential variables with *p* < 0.10 in the univariable analysis or thought to be independent predictors of ischemic stroke were entered into a multivariable LR with a backward stepwise. Variables with *p* < 0.05 were considered statistically significant, and all p were two-sided. Finally, our models were developed based on ML, including age, premorbid mRS, NIHSS at admission, creatinine, and FBG. All statistical analyses were performed using SPSS version 22.0 (IBM Corporation, Armonk, NY, USA) and Stata version 13.0 (StataCorp, College Station, TX, USA).

### Model Development

According to Wolpert's “No Free Lunch Theorem,” no one technique will be most accurate in every case, and so comparisons of techniques in different research areas and datasets may yield different results (15). Therefore, we used 5 ML algorithms: LR, SVM, RFC, XGBoost, and DNN because they are widely and successfully used for clinical data (16–20).

As a standard way of estimating the performance of the model, the k-fold cross-validation method is more reliable than simply holding out the validation set by giving the variance of the performance and has been used in various reports (16–19). The 5-fold cross-validation was used for the model derivation and internal evaluation by dividing the training set into five mutually exclusive parts, four of which were used as training data to generate the model and one for evaluation as inner validation data; this process was repeated five times to generate five different but overlapping training data and five unique validation data. Due to the long training time and high resource consumption of DNN, we used a random partition of 10% data as a validation set instead of 5-fold cross-validation to optimize the model. In the training step, we optimized model hyperparameters with a grid search algorithm. During the searching process, we set the area under the curve (AUC) of receiver operating characteristic (ROC) as the score.

### Model Evaluation

After the models were derived, the sensitivity, specificity, accuracy, and AUC were calculated for the testing data. The performances of different models were compared by ROC analysis and Delong test.

For evaluating the superiority of prediction capability for the ML models, we calculated THRIVE score, HIAT score, and NADE nomogram on the same patient group. Although there were some other scores, they were not included because the database lacked information for the calculation (21, 22). In addition, we also developed 2 ML models (LR and RFC models) using 21 variables with *p* < 0.10 in a univariable analysis as a reference. After derivation of the models, we calculated the contribution of each variable: the absolute value of the standardized regression coefficient for LR and information gain (which was estimated by the decrease in impurity) for RFC. The five ML models were developed and validated with open-source packages in Python software (version 3.7): Scikit-learn, keras, and XGboost.

## Results

### Patient Characteristics

**Figure 1:**
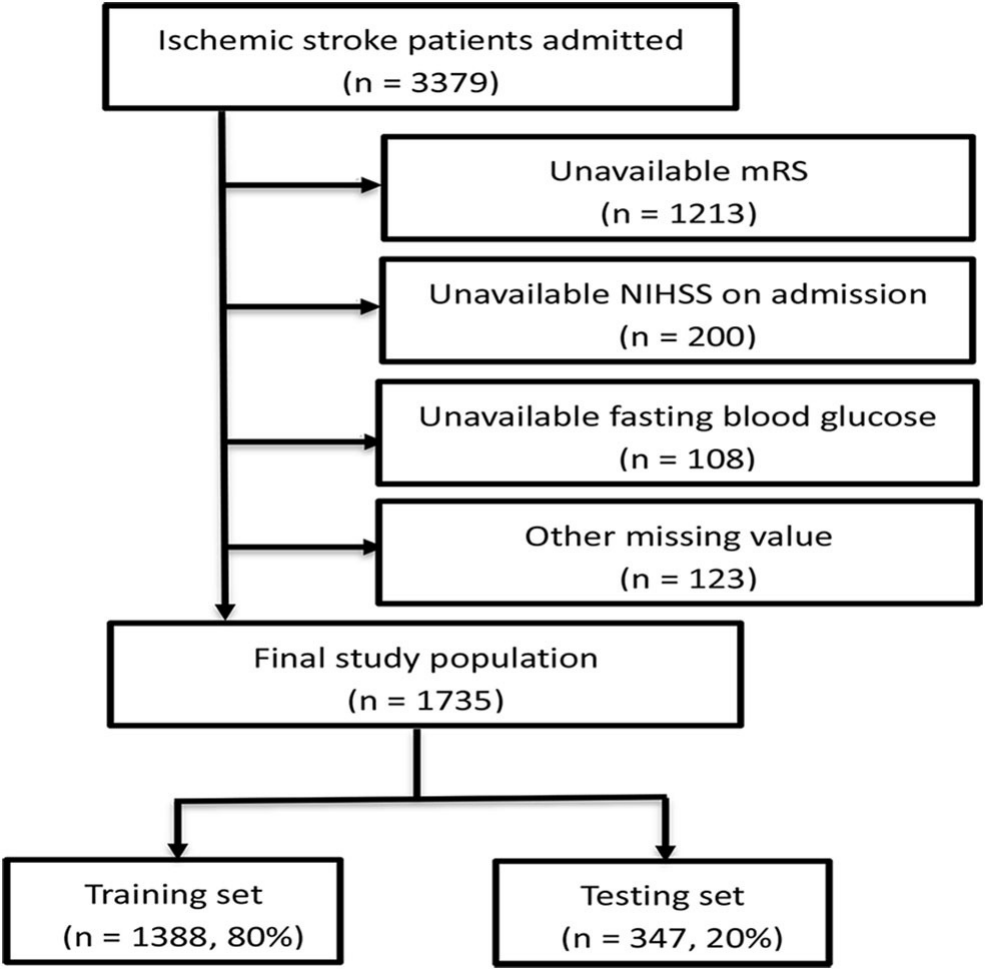
Flow chart illustrating patient selection. mRS, modified Rankin Scale; NIHSS, National Institute of Health stroke scale.

A total of 3,379 patients were registered to the cohort during the study period. After excluding 1,213 patients with unavailable 6-month mRS scores, 200 patients with unavailable NIHSS at admission, 108 patients with unavailable FBG, and 123 patients with missing other laboratory tests or clinical data, 1,735 patients were finally included (Figure 1). Comparison of demographic variables between the included and excluded patients is shown in Supplementary Table 1. The median age of the 1,735 patients was 68 (IQR:60–78) years, and 67.1% were men. The proportion of patients with unfavorable outcome was 31.2% (541/1,735), and 12.0% (208/1,735) died within the follow-up period (mRS score = 6). The characteristics of the patients were well-balanced between the training (*n* = 1,388, 80%) and testing (*n* = 347, 20%) sets (Supplementary Table 2).

### Feature Selection

The 21 variables with *p* < 0.10 in the univariable analysis or thought to be independent predictors of ischemic stroke (the variables list is shown under Table 1) entered into the LR. After multivariate LR analysis, age, NIHSS at admission, premorbid mRS, FBG, and creatinine remained independent predictors of 6-month unfavorable outcome.

**Table 1:** Clinical, demographic and laboratory data of the patients in the training set stratified according to 6-month favorable or unfavorable outcome after acute ischemic stroke.

	**Favorable outcome (mRS 0–2)**	**Unfavorable outcome (mRS 3–6)**	***P*-value**
Patients, n	955	433	
Age, years, median (IQR)	66 (58–74)	77 (68–83)	<0.0001#*
Sex, n (%)			<0.0001*
Male	682 (71.4)	254 (58.7)	
Female	273 (28.6)	179 (41.3)	
Onset-to-admission delay <4.5 h, n (%)	236 (24.7)	127 (29.3)	0.070*
Medical history, n (%)			
Hypertension	650 (68.1)	317 (73.2)	0.053*
Diabetes mellitus	248 (26.0)	143 (33.0)	0.007*
Hyperlipidemia	29 (3.0)	6 (1.4)	0.069*
Coronary artery disease	100 (10.5)	77 (17.8)	<0.0001*
Atrial fibrillation	75 (7.9)	95 (21.9)	<0.0001*
Previous cerebral infarction	123 (12.9)	102 (23.6)	<0.0001*
Valvular heart disease	13 (1.4)	8 (1.8)	0.492
Smoking, n (%)			<0.0001*
Never smoker	394 (41.3)	255 (58.9)	
Former smoker	129 (13.5)	66 (15.2)	
Current smoker	432 (45.2)	112 (25.9)	
Drinking, n (%)			<0.0001*
Never drinker	525 (55.0)	307 (70.9)	
Former drinker	84 (8.8)	47 (10.9)	
Current drinker	346 (36.2)	79 (18.2)	
Baseline data			
Premorbid mRS, median (IQR)	0 (0–0)	0 (0–2)	<0.0001#*
NIHSS at admission, median (IQR)	3 (2–5)	10 (5–16)	<0.0001#*
BMI, kg/m^2^, median (IQR)	24.38 (22.38–26.64)	24.03 (21.60–26.37)	0.046#*
Pulse, times/min, median (IQR)	76 (70–80)	76 (70–84)	0.005#*
Systolic BP, mmHg, median (IQR)	140 (130–160)	142 (130–160)	0.350#
Diastolic BP, mmHg, median (IQR)	84 (80–94)	83 (78–95)	0.533#
Platelet count, 10^9^/L, median (IQR)	195 (159–234)	188 (150–238)	0.159#
Urea nitrogen, mmol/L, median (IQR)	5.23 (4.4–6.34)	6.12 (4.71–7.75)	<0.0001#*
Creatinine, μmol/L, median (IQR)	71 (59–83)	76 (62–97)	<0.0001#*
FBG, mmol/L, median (IQR)	5.08 (4.50–6.21)	6.40 (5.05–7.99)	<0.0001#*
TC, mmol/L, median (IQR)	4.41 (3.76–5.16)	4.41 (3.64–5.18)	0.574#
TG, mmol/L, median (IQR)	1.31 (0.96–1.86)	1.18 (0.84–1.59)	<0.0001#*
LDL, mmol/l, median (IQR)	2.71 (2.13–3.31)	2.76 (1.96–3.27)	0.470#
HDL, mmol/l, median (IQR)	1.05 (0.9–1.23)	1.08 (0.9–1.26)	0.165#
Endovascular therapy, n (%)	72 (7.5)	55 (12.7)	0.002*
IV thrombolysis, n (%)	208 (21.8)	119 (27.5)	0.020*

*mRS, modified Rankin Scale; IQR, interquartile range; NIHSS, National Institute of Health stroke scale; BMI, body mass index; BP, blood pressure; FBG, fasting blood glucose; TC, total cholesterol; TG, triglyceride; LDL, low-density lipoprotein; HDL, high-density lipoprotein; IV, intravenous*.

# *calculated using Mann-Whitney U-test*.

* *included into the multiple logistic regression models (P < 0.1)*.

### Model Performance

**Figure 2:**
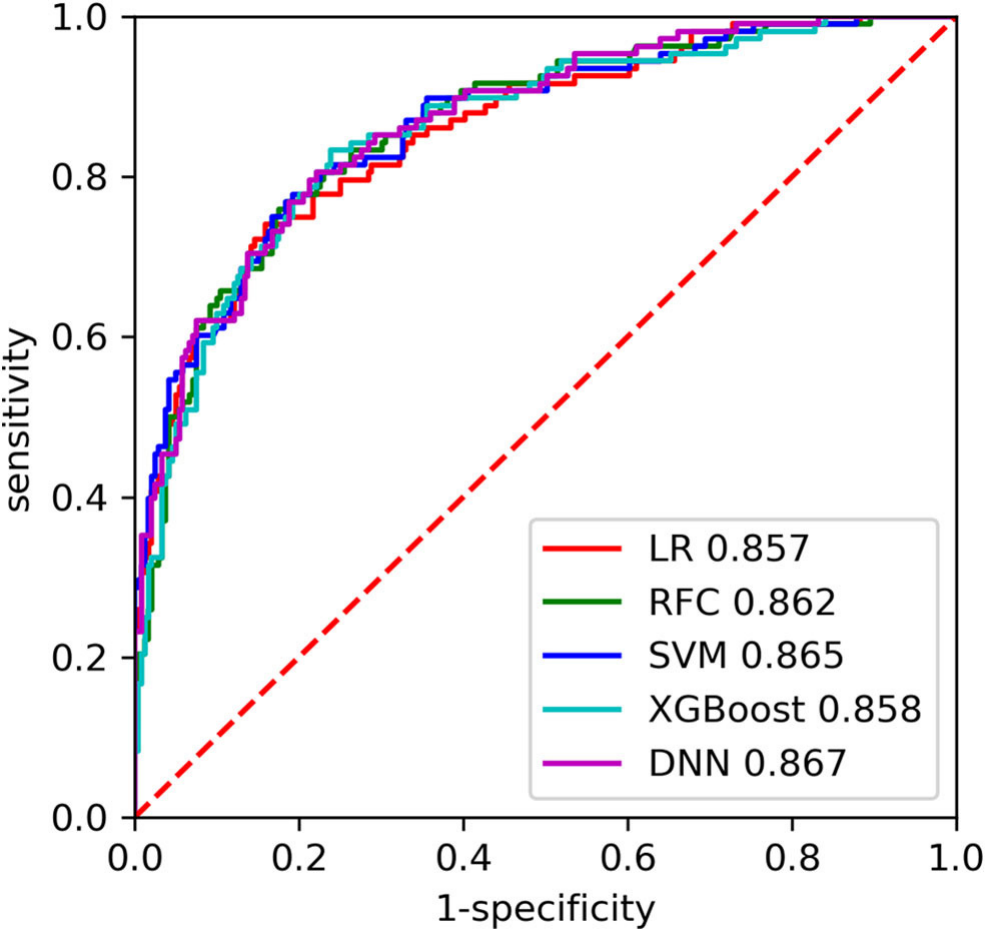
The receiver operating characteristic (ROC) curves of the machine learning (ML) models on the testing set. LR, logistic regression; SVC, support vector machine; RFC, random forest classifier; XGBoost, extreme gradient boosting; DNN, fully-connected deep neural network.

The AUC of each model on the training and inner validation sets is provided in Table 2. The AUC of each model on the testing set is given as follows (Table 3, Figure 2): LR 0.857 [95% CI, 0.814–0.900], SVM 0.865 [0.823–0.907], RFC 0.862 [0.820–0.904], XGBoost 0.858 [0.815–0.901], and DNN 0.867 [0.827–0.908]. *P-*values for AUC of RFC compared with LR, SVM, XGBoost, and DNN were 0.885, 0.930, 0.898, and 0.848, respectively. Although there was no difference in AUC between the ML models, we chose the RFC model to compare with previously reported models.

**Table 2:** The area under the curve (AUC) of training set and inner validation set.

**Models**	**Training set (95% CI)**	**Inner validation set (95% CI)**
LR	0.867 (0.847–0.888)	0.862 (0.812–0.911)
SVM	0.874 (0.855–0.894)	0.871 (0.840–0.901)
RFC	0.897 (0.880–0.915)	0.866 (0.831–0.902)
XGBoost	0.890 (0.872–0.908)	0.867 (0.833–0.901)
DNN	0.877 (0.858–0.897)	0.860 (0.825–0.896)
LR*	0.874 (0.853–0.894)	0.865 (0.833–0.897)
RFC*	0.899 (0.881–0.917)	0.865 (0.835–0.894)

*LR, logistic regression; SVM, support vector machine; RFC, random forest classifier; XGBoost, extreme gradient boosting; DNN, fully-connected deep neural network*.

* *indicates model developed with 21 variables*.

**Table 3:** Scores for each model in the testing set.

**Models**	**AUC (95% CI)**	**Specificity**	**Sensitivity**	**Precision**	**Accuracy**
LR	0.857 (0.814–0.900)	0.912	0.620	0.761	0.821
SVM	0.865 (0.823–0.907)	0.912	0.602	0.756	0.816
RFC	0.862 (0.820–0.904)	0.883	0.657	0.717	0.813
XGBoost	0.858 (0.815–0.901)	0.895	0.630	0.731	0.813
DNN	0.867 (0.827–0.908)	0.891	0.556	0.811	0.821
LR*	0.866 (0.825–0.907)	0.921	0.593	0.780	0.821
RFC*	0.874 (0.835–0.912)	0.950	0.500	0.818	0.810

*AUC, the area under the curve; LR, logistic regression; SVM, support vector machine; RFC, random forest classifier; XGBoost, extreme gradient boosting; DNN, fully-connected deep neural network*.

* *indicates model developed with 21 variables*.

**Figure 3:**
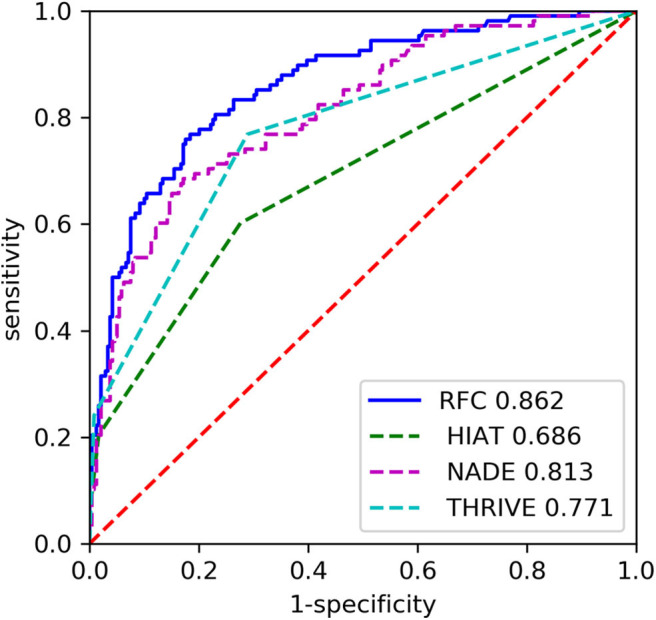
The receiver operating characteristic (ROC) curves of the random forest classifier (RFC) and previous models on the testing set. HIAT, Houston Intra-Arterial Therapy score; THRIVE, Totaled Health Risks in Vascular Events score; NADE, NIHSS score on admission, age, previous diabetes mellitus and creatinine.

The RFC model performed significantly better than the THRIVE score [AUC 0.862 [0.820–0.904] vs. 0.771 [0.721–0.822]; *p* = 0.007], HIAT score [AUC 0.862 [0.820–0.904] vs. 0.686 [0.630–0.743]; *p* < 0.0001], and NADE nomogram [AUC 0.862 [0.820–0.904] vs. 0.813 [0.763–0.862]; *p* = 0.011] (Figure 3). The sensitivity of RFC model is 0.657, while specificity is 0.883.

**Figure 4:**
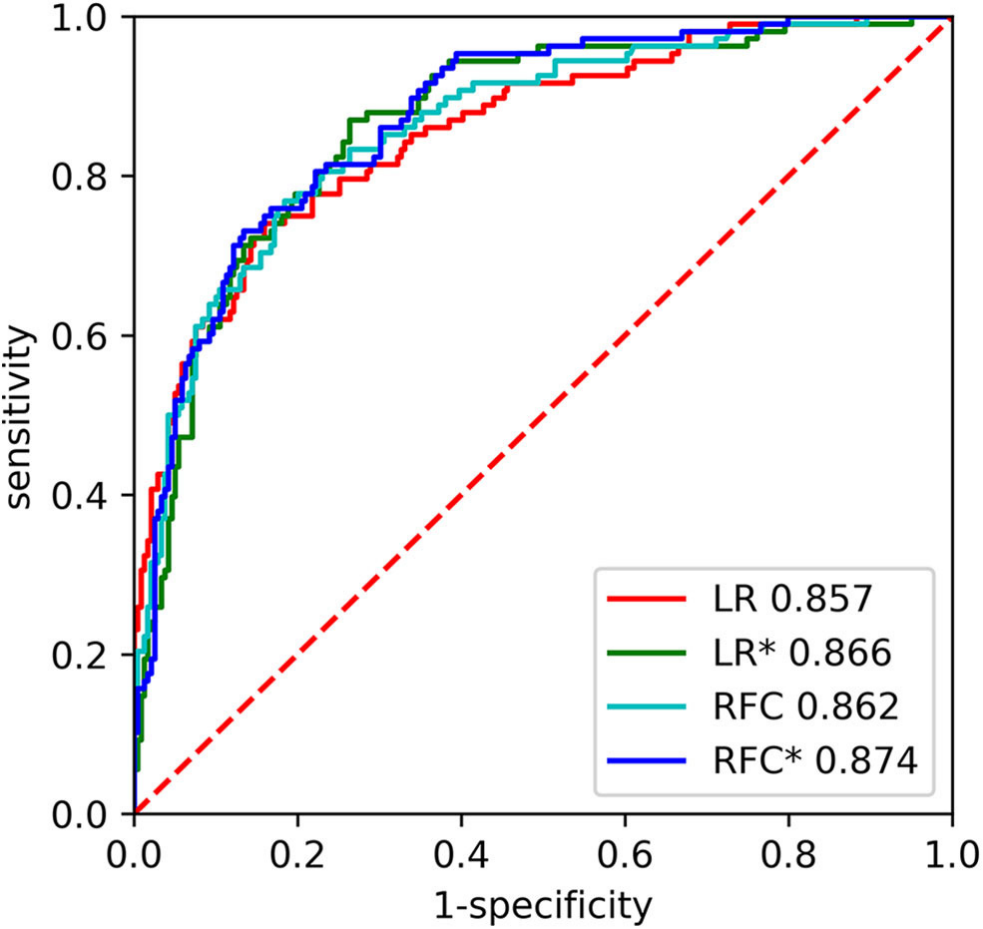
The receiver operating characteristic (ROC) curves of the random forest classifier (RFC) and logistic regression (LR) on the testing set. * indicates model developed with 21 variables.

After we developed LR and RFC model with 21 variables, the performance of ML models did not differ from these models with five variables [0.857 [0.814–0.900] vs. 0.866 [0.825–0.907], *p* = 0.755 and 0.862 [0.820–0.904] vs. 0.874 [0.835–0.912], *p* = 0.665] (Table 3, Figure 4). Furthermore, we calculated the six most important variables in LR and RFC model using 21 variables. Age, NIHSS at admission, premorbid mRS, FBG, and creatinine also appeared as the most important variables (Table 4).

**Table 4:** Top 6 important features in the models with 21 variables.

**NO**.	LR*	RFC*
1	NIHSS at admission	NIHSS at admission
2	Premorbid mRS	Age
3	Age	Premorbid mRS
4	Fasting blood glucose	Fasting blood glucose
5	Creatinine	Urea nitrogen
6	Sex	Creatinine

*LR, logistic regression; RFC, random forest classifier; NIHSS, National Institute of Health stroke scale; mRS, modified Rankin Scale*.

* *indicates model developed with 21 variables*.

## Discussion

To the best of our knowledge, this is the first study that develops prediction models with ML methods for the 6-month clinical outcome of AIS patients. For predicting 6-month unfavorable functional outcome in Chinese AIS patients, our study suggested that the RFC model is more accurate than the HIAT score, the THRIVE score, and the NADE nomogram. End users in clinical practice will be able to perform more accurate evaluation of long-term outcome for AIS patients when our models are fed five easily accessible variables. Thus, it interests clinical physicians because of importance of outcome prediction for patients and their families. Although, there are some criticisms about ML techniques because they are black boxes (23). Importantly, our model should be used together with, rather than instead of, clinical judgment. Combining machines plus physicians reliably enhances system performance. Hence, we should strongly consider the RFC model if accuracy is paramount.

As a popular ensemble method, RFC has been successfully applied in medical fields due to its ability to build predictive models with high certainty and little necessity of model optimization. In particular, the important advantages were shown in the RFC model compared with other methodologies, including the ability to handle highly non-linearly correlated data, robustness to noise, and tuning simplicity (24). In our research, some strategies to avoid overfitting were performed, and our results showed no signs of obvious overfitting in the RFC model. Additionally, to ensure an unbiased and robust performance, 5-fold cross-validation was iteratively used. The preceding characteristic features may make our model useful in real-world practice.

Several previous prognostic models have been developed to predict the clinical outcome after stroke, such as the HIAT score, the THRIVE score, and the NADE nomogram (3–5). The HIAT score identified three predictors of a 3-month unfavorable outcome in intra-arterial recanalization therapy, that is age > 75 years, NIHSS > 18, and baseline glucose level ≥ 150 mg/dL. It respectively, has an AUC value of 0.69 and 0.73 in two cohorts (3). The THRIVE score identified age, NIHSS, hypertension, diabetes mellitus, and atrial fibrillation are independently associated with a 3-month poor outcome (4). It was well-validated with a large sample size from the Virtual International Stroke Trials Archive and had an AUC of 0.75 (25). The NADE nomogram was developed to predict 6-month unfavorable outcome after AIS (5). NIHSS at admission, age, previous diabetes mellitus, and creatinine were found to be significant predictors. The AUC value of the NADE nomogram was 0.791. In our study, only five variables—age, NIHSS on admission, premorbid mRS, FBG, and creatinine—were included into our models. NIHSS and premorbid mRS indicated that stroke severity and degree of dependence in the daily activities influenced the stroke outcome. Blood glucose has been proven to be not only associated with stroke outcome but also a risk factor of symptomatic intracerebral hemorrhage after thrombolysis therapy (26). Creatinine is an indicator of renal function. However, the relationship between renal function and stroke outcomes is controversial (27, 28). Indeed, after excluding some less important and even misleading variables for stroke outcome, ML models based on 5 and 21 variables have achieved similar performance. This illustrates that the five variables we selected contained almost all useful information in the original data. In addition, variable importance derived from the RFC and LR with 21 variables also provides insight for the importance of individual variables in prediction performance.

There are some limitations in our study. First, our population was from a single hospital. The generalizability of the predictive models needs to be tested in patients treated at other institutions and by other surgeons. Second, there were some cases that were lost to follow-up. Especially, of total 1,213 cases excluded from this study for loss of 6-month follow-up, 944 cases (78%) were lost between May 2017 and March 2018, accounting for 85% of the AIS patients during that period. It is unclear whether we have overestimated or underestimated the unfavorable outcome after AIS. But we believe this centralized loss of data may have less impact on the results. Finally, data of known neurobiological predictors of 3-month outcome such as infarct size (29) were not available in our study. Future prospective studies will have to assess whether incorporating novel predictors may improve the accuracy of predictive model.

## Conclusions

To sum up, the comparison with the previous models demonstrated that it is feasible to apply the RFC model to stroke patient management, which achieves optimal performance compared with the HIAT score, THRIVE score, and NADE nomogram. Moreover, the RFC model is easy to use and robust. These advanced characteristics may contribute to reliable and practical applications in clinical practice.

## Data Availability Statement

The datasets generated for this study are available on request to the corresponding author.

## Ethics Statement

Approval from the ethics committee of Nanjing First Hospital was obtained.

## Author Contributions

XL and JZo concepted, designed, and supervised the study. JZh, YL, FW, XZ, JY, and YZ acquired the data. XL and ZZ analyzed and interpreted the data, provided statistical analysis, had full access to all of the data in the study, and are responsible for the integrity of the data and the accuracy of the data analysis. XL, XP, MW, CS, ZZ, and SW drafted the manuscript. JZo and CJ critically revised the manuscript for important intellectual content. All authors contributed to the article and approved the submitted version.

## Conflict of Interest

The authors declare that the research was conducted in the absence of any commercial or financial relationships that could be construed as a potential conflict of interest.
