# Supplementary material for: Predicting 6-Month Unfavorable Outcome of Acute Ischemic Stroke Using Machine Learning
Source: Front Neurol. 2020 Nov 19;11:539509. doi: 10.3389/fneur.2020.539509 (PMC7710984; doi:10.3389/fneur.2020.539509)
Supplement: Supplementary file 1 [file Data_Sheet_1.docx]

**TABLE S1** Demographics and clinical characteristics of the patients excluded and included.

|  | **Excluded patients**  **(n=1644)** | **Included patients**  **(n=1735)** |
| --- | --- | --- |
| Age, years, median (IQR) | 68(60-76) | 68(60-78) |
| Male sex, n (%) | 1055(66.1) | 1164(67.1) |
| Onset-to-admission delay <4.5h, n (%) | 372(26.2) | 466(26.9) |
| Premorbid mRS, pts, median (IQR) | 0(0-0) | 0(0-0) |
| Medical history, n (%) |  |  |
| Hypertension | 1170(72.6) | 1212(69.9) |
| Diabetes mellitus | 465(28.9) | 470(27.1) |
| Hyperlipidemia | 46(2.9) | 52(3.0) |
| Coronary artery disease | 201(12.5) | 228(13.1) |
| Atrial fibrillation | 163(10.1) | 222(12.8) |
| Previous cerebral infarction | 343(21.3) | 297(17.1) |
| Valvular heart disease | 23(1.4) | 33(1.9) |
| Smoking, n (%) |  |  |
| Never smoker | 771(48.2) | 809(46.6) |
| Former smoker | 245(15.3) | 242(13.9) |
| Current smoker | 585(36.5) | 684(39.4) |
| Drinking, n (%) |  |  |
| Never drinker | 939(58.7) | 1031(59.4) |
| Former drinker | 201(12.6) | 175(10.1) |
| Current drinker | 459(28.7) | 529(30.5) |
| Baseline data |  |  |
| NIHSS at admission, median (IQR) | 3(2-8) | 4(2-9) |
| BMI, kg/m^2^, median (IQR) | 24.22(22.22-26.64) | 24.22(22.04-26.57) |
| pulse, times/min, median (IQR) | 76(70-78) | 76(70-80) |
| Systolic BP, mmHg, median (IQR) | 140(130-154) | 141(130-160) |
| Diastolic BP, mmHg, median (IQR) | 80(78-90) | 83(80-94) |
| Platelet count, 10^9^/L, median (IQR) | 192(156-233) | 193(157-234) |
| Urea nitrogen, mmol/L, median (IQR) | 5.5 (4.55-6.71) | 5.5(4.5-6.75) |
| Creatinine, μmol/L, median (IQR) | 70(59-85) | 73(60-87) |
| FBG, mmol/L, median (IQR) | 5.24(4.56-6.66) | 5.33(4.64-6.87) |
| TC, mmol/L, median (IQR) | 4.21(3.55-4.97) | 4.38(3.69-5.15) |
| TG, mmol/L, median (IQR) | 1.24(0. 90-1.82) | 1.27(0.91-1.79) |
| LDL, mmol/l, median (IQR) | 2.50(1.94-3.11) | 2.67(2.05-3.27) |
| HDL, mmol/l, median (IQR) | 1.05(0.89-1.24) | 1.05(0.89-1.23) |
| Endovascular therapy, n (%) | 197(12.1) | 156(9.0) |
| IV thrombolysis, n (%) | 220(13.5) | 418(24.1) |
| mRS 3–6 at 6 months, n (%) | 106(24.6) | 541(31.2) |

mRS, modified Rankin Scale; IQR, interquartile range; NIHSS, National Institute of Health stroke scale; BMI, body mass index; BP, blood pressure; FBG, fasting blood glucose; TC, total cholesterol; TG, triglyceride; LDL, low density lipoprotein; HDL, high-density lipoprotein; IV, intravenous.

**TABLE S2** Demographics and clinical characteristics of the patients in the training and testing set

|  | **Training set**  **(n=1388)** | **Testing set**  **(n=347)** | **p** |
| --- | --- | --- | --- |
| Age, years, median (IQR) | 69(60-78) | 68(60-76) | 0.474# |
| Sex, n (%) |  |  | 0.540 |
| Male | 936(67.4) | 228(65.7) |  |
| Female | 452(32.6) | 119(34.3) |  |
| Onset-to-admission delay <4.5h, n (%) | 363(26.2) | 103(29.7) | 0.184 |
| Premorbid mRS, pts, median (IQR) | 0(0-0) | 0(0-0) | 0.986 |
| Medical history, n (%) |  |  |  |
| Hypertension | 967(69.7) | 245(70.6) | 0.734 |
| Diabetes mellitus | 391(28.2) | 79(22.8) | 0.043 |
| Hyperlipidemia | 35(2.5) | 17(4.9) | 0.020 |
| Coronary artery disease | 177(12.8) | 51(14.7) | 0.337 |
| Atrial fibrillation | 170(12.2) | 52(15.0) | 0.172 |
| Previous cerebral infarction | 225(16.2) | 72(20.7) | 0.045 |
| Valvular heart disease | 21(1.5) | 12(3.5) | 0.018 |
| Smoking, n (%) |  |  | 0.919 |
| Never smoker | 649(46.8) | 160(46.1) |  |
| Former smoker | 195(14.0) | 47(13.5) |  |
| Current smoker | 544(39.2) | 140(40.3) |  |
| Drinking, n (%) |  |  | 0.197 |
| Never drinker | 832(59.9) | 199(57.3) |  |
| Former drinker | 131(9.4) | 44(12.7) |  |
| Current drinker | 425(30.6) | 104(30.0) |  |
| Baseline data |  |  |  |
| NIHSS at admission, median (IQR) | 4(2-9) | 4(2-9) | 0.836# |
| BMI, kg/m^2^, median (IQR) | 24.22(22.08-26.57) | 24.22(22.04-26.67) | 0.484# |
| pulse, times/min, median (IQR) | 76(70-80) | 76(70-80) | 0.752# |
| Systolic BP, mmHg, median (IQR) | 141(130-160) | 141(130-160) | 0.673# |
| Diastolic BP, mmHg, median (IQR) | 83(80-94) | 82(78-92) | 0.306# |
| Platelet count, 10^9^/L, median (IQR) | 192(157-235) | 194(158-233) | 0.995# |
| Urea nitrogen, mmol/L, median (IQR) | 5.40(4.48-6.72) | 5.70(4.51-6.9) | 0.112# |
| Creatinine, μmol/L, median (IQR) | 72.7(60.0-86.0) | 73.0(60.0-90.1) | 0.489# |
| FBG, mmol/L, median (IQR) | 5.31(4.62-6.91) | 5.37(4.70-6.68) | 0.694# |
| TC, mmol/L, median (IQR) | 4.41(3.74-5.17) | 4.21(3.56-4.96) | 0.049# |
| TG, mmol/L, median (IQR) | 1.27(0. 92-1.78) | 1.27(0.91-1.86) | 0.676# |
| LDL, mmol/l, median (IQR) | 2.72(2.08-3.29) | 2.51(1.95-3.20) | 0.131# |
| HDL, mmol/l, median (IQR) | 1.06(0.9-1.24) | 1.04(0.87-1.22) | 0.021# |
| Endovascular therapy, n (%) | 127(9.1) | 29(8.4) | 0.644 |
| IV thrombolysis, n (%) | 327(23.6) | 91(26.2) | 0.433 |
| mRS 3–6 at 6 months, n (%) | 433(31.2) | 108(31.1) | 0.979 |

# Calculated using Mann-Whitney U test.

mRS, modified Rankin Scale; IQR, interquartile range; NIHSS, National Institute of Health stroke scale; BMI, body mass index; BP, blood pressure; FBG, fasting blood glucose; TC, total cholesterol; TG, triglyceride; LDL, low density lipoprotein; HDL, high-density lipoprotein; IV, intravenous.

**TABLE S3** Hyper-parameters of ML-based models.

| **Model** | **Hyper-parameters** | **Value** |
| --- | --- | --- |
| LR | C | 0.3 |
|  | Penalty | L2 |
| SVM | C | 0.5 |
|  | Gamma | 0.035 |
|  | Kernel | rbf |
| RFC | N_estimators | 85 |
|  | Criterion | gini |
|  | Max_depth | 5 |
|  | Max_leaf_nodes | 13 |
| XGB | Max_depth | 4 |
|  | Learning_rate | 0.04 |
|  | N_estimators | 50 |
|  | Min_child_weight | 5 |
|  | Gamma | 0.8 |
|  | Subsample | 0.35 |
|  | Colsample_bytree | 0.85 |
| DNN | Hidden layers | 12-8-4 |
|  | Dropout | 0.5 |
|  | Optimizer | Adam |
|  | Initial learning rate | 0.001 |
|  | Activation | Hidden layers: Relu; output layer: sigmoid |
|  | Loss functiuon | binary_crossentropy |
|  | Epochs | 200 |

*LR, logistic regression; SVM, support vector machine; RFC, random forest classiﬁer; XGBoost, extreme gradient boosting; DNN, fully-connected deep neural network.*
